# Abbreviating the Wealth Index to Measure Equity in Health Programs More Easily

**DOI:** 10.9745/GHSP-D-16-00028

**Published:** 2016-03-25

**Authors:** Thomas W Pullum

**Affiliations:** a ICF International, The Demographic and Health Surveys Program, Rockville, MD, USA

## Abstract

Efforts to simplify the construction of the DHS wealth index are encouraged (while recognizing it is constructed differently in each country), but attempts to assess equity in health programs should bear in mind that it is not sufficient to calculate the wealth index just for the participants in the program. The quintile distributions can vary dramatically within sub-populations. Assessments of equity require knowledge of the distribution of potential participants as well as actual participants.

See related articles by Chakraborty and by Ergo

This issue of *Global Health: Science and Practice* (GHSP) includes 2 articles—one by Chakraborty and colleagues^1^ and the other by Ergo and colleagues^2^—that describe how the well-known Demographic and Health Surveys (DHS) wealth index can be adapted for the purpose of assessing equity in health programs. DHS constructs the index by combining information about a large number of household assets in a principal components analysis, interpreting the first principal component as a continuous single dimension of wealth, and then identifying cut-points that break that scale into 5 segments, known as wealth quintiles.

The strategies used to adapt the DHS wealth index in the 2 GHSP articles are different from each other, but both are able to produce a good approximation to the wealth index and wealth quintiles with a much smaller number of indicators. In the article by Ergo et al., the original wealth index is trimmed by dropping the indicators with the smallest loadings in successive recalculations of the first principal component. In the article by Chakraborty et al., the indicators are trimmed by a combination of statistical criteria and judgments by a panel of experts. Realizing that the DHS wealth index is constructed differently in each country, both strategies could be applied to a country that had recently had a DHS survey (or Multiple Indicator Cluster Survey [MICS]), working with the household data files and complete information about how the wealth index was constructed in that survey, including the numerical values of the cut-points.

The authors of the 2 GHSP articles propose that the smaller set of indicators could be obtained with a much-reduced questionnaire that would be administered to the beneficiaries of an intervention, and then the simplified wealth quintiles would be constructed to determine the degree of equity in the intervention. Neither paper actually provides a summary index of equity, but both imply that an intervention would be equitable if the number of beneficiaries were the same, or nearly the same, in all 5 quintiles.

## Some Caveats: Wealth Quintile Distribution Varies Across Sub-Populations and Comparisons Across Quintiles Need to Account for Both Beneficiaries and *Potential* Beneficiaries

My purpose here is not to critique these constructions of a simplified wealth index, but to set up a warning to the authors or others who may apply it to assess the equity of interventions. The distribution across wealth quintiles is uniform for a DHS survey, in the sense that there will be exactly the same number of weighted usual resident individuals in the full household sample in each of the 5 quintiles. Deviations from 20% in each quintile are negligible and are only due to possible ties at specific values of the continuous scale. For virtually any *sub-population*, however, the wealth quintiles *do not* identify 5 equally-sized categories. For example, in most DHS surveys, few urban respondents are in the bottom 2 quintiles and few rural respondents are in the top 2 quintiles. In many surveys, more than 20% of children under age 18 are in the bottom quintile and more than 20% of adults are in the top quintile, because of the tendency for poorer households to have more children. The distribution across quintiles can vary enormously from one region of a country to another.

Further, it is impossible to assess equity in an intervention by examining only the distribution of the *beneficiaries* across the 5 quintiles. A valid assessment of equity would also require knowledge of the distribution of the *potential* beneficiaries, so that a comparison can be made of the differences between the people who use the program and those who are eligible, or are in the catchment area, etc., but do not use the program. For a familiar analogy, it is impossible to determine whether a college admissions process has been fair with respect to ethnicity by knowing only the ethnic distribution of the acceptances. Fairness, or equity, would require knowledge of the ethnic distribution of the pool of applicants, so that a comparison could be made between those who were accepted and those who were rejected. The ethnic distribution of applicants could not safely be assumed to be the same as the ethnic distribution of the general population.

An alternative way to assess equity is to measure the *level of an attribute within quintiles* and then compare those levels. This approach is illustrated in the GHSP article by Jorge Ugaz et al.^3^ One outcome of interest in that article is the percentage of users of modern methods of contraception who are using long-acting methods. This percentage is reported within each of the wealth quintiles. Data come from several DHS surveys, using the original wealth index constructed for each survey. Variations in the percentages across wealth quintiles can be interpreted as evidence of inequities in access to long-acting methods. Similarly, in the college admissions analogy, variations in acceptance rates from one ethnic group of applicants to another can be interpreted as a lack of fairness in the admissions procedure.

Improving equity in programs is a highly worthy goal. Efforts such as those by Ergo et al. and Chakraborty et al. to simplify the measurement of relative wealth and equity are commendable, but an assessment of equity in health interventions requires information about those who access the intervention and those who are eligible but do not access it. In one way or another, any application of a simplified wealth index to assess equity must confront this requirement.

Assessment of equity in health interventions requires information about those who access the intervention as well as those who are eligible but do not access it.
